# Correction: Development and testing of a composite index to monitor the continuum of maternal health service delivery at provincial and district level in South Africa

**DOI:** 10.1371/journal.pone.0352153

**Published:** 2026-06-22

**Authors:** Mamothena Carol Mothupi, Jeroen De Man, Hanani Tabana, Lucia Knight

In Fig 3, the Y axis is labelled “CCoC index.” The Y axis should be labelled “C_3_MH index.” Please see the correct Fig 3 and caption here.

In Fig 4, the Y axis contains the label “COC index.” The Y axis should display the label “C_3_MH index.” Please see the correct Fig 4 and caption here.

**Fig 3:**
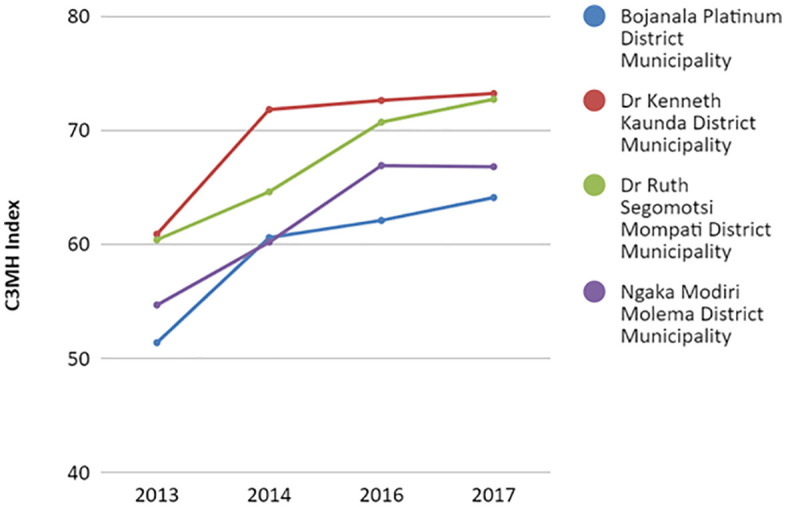
Comprehensive continuum of care for maternal heatlh index (C_3_MH index) scores by districts over a five-year period 2013–2017.

**Fig 4:**
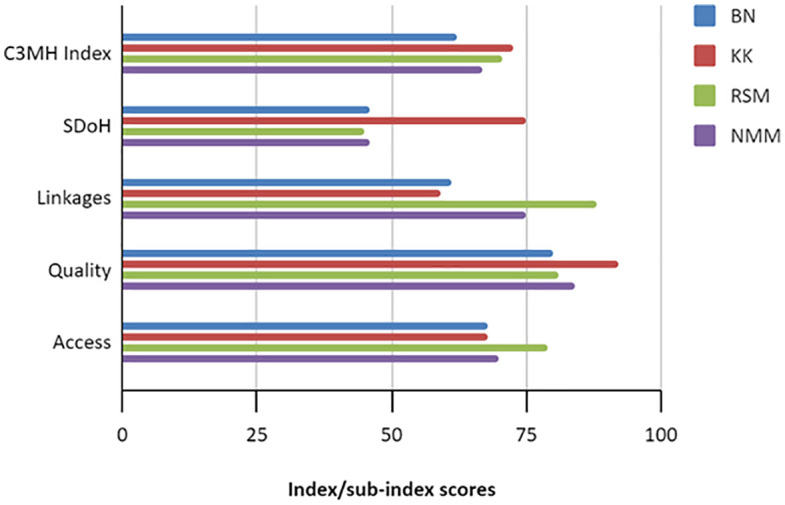
Sub-index and C_3_MH index scores by districts in 2016. BN = Bojanala Platinum District, KK = Dr Kenneth Kaunda District Municipality, RSM = Dr Ruth Segomotsi Mompati District Municipality, NMM = Ngaka Modiri Molema District Municipality, SDOH = Social determinants of health.
