# Supplementary material for: Development and testing of a composite index to monitor the continuum of maternal health service delivery at provincial and district level in South Africa
Source: PLoS One. 2021 May 25;16(5):e0252182. doi: 10.1371/journal.pone.0252182 (PMC8148336; doi:10.1371/journal.pone.0252182)
Supplement: S1 Table — (DOCX) [file pone.0252182.s001.docx]

|  | **Indicators** | **Definition** | **Source** |
| --- | --- | --- | --- |
| 1 | Cervical cancer screening coverage | Cervical smears in women 20 years and older as a proportion of the target for cervical cancer screening. Females over 20 years of age who are HIV-positive must be screened for cervical cancer every 3 years and others screened every 10 years. | NIDS |
| **2** | Antenatal 1st visit before 20 weeks rate | Women who have a booking visit (first visit) before they are 20 weeks into their pregnancy as proportion of all antenatal 1st visits | NIDS |
| **3** | Antenatal 1st visit coverage | The proportion of potential antenatal clients coming for at least one (booking) antenatal visit. The census number of children under one year factorised by 1.15 is used as a proxy denominator - the extra 0.15 (15%) is a rough estimate to cater for late miscarriages (~10 to 26 w), stillbirths (after 26 weeks gestation) and infant mortality. Pregnant women are regarded as potential antenatal clients from around 10 weeks’ gestation, i.e. spontaneous abortions before that as well as ToP cases are excluded | NIDS |
| **4** | Syphilis positive pregnant female receive Benz-penicillin 1st dose rate | Syphilis positive pregnant females who received Benz-penicillin 1st dose as a proportion of pregnant females who tested positive for syphilis | DHIS |
| **5** | Syphilis positive pregnant female receive Benz-penicillin 2nd dose rate | Syphilis positive pregnant female receive Benz-penicillin 2nd dose as a proportion of pregnant females who tested positive for syphilis | DHIS |
| **6** | Syphilis positive pregnant female receive Benz-penicillin 3rd dose rate | Syphilis positive pregnant female receive Benz-penicillin 3rd dose as a proportion of pregnant females who tested positive for syphilis | DHIS |
| **7** | Antenatal client start on ART rate | Antenatal clients who started on ART as a proportion of the total number of antenatal clients who are HIV positive and not previously on ART |  |
| **8** | Delivery in facility rate | Deliveries in health facilities as proportion of expected deliveries in the population. Expected deliveries are estimated as population under 1 year multiplied by 1.025 to compensate for still births and infant mortality | NIDS |
| **9** | Delivery by caesarean section rate | Delivery by Caesarean section as proportion of total deliveries in health facilities | NIDS |
| **10** | Mother postnatal visit within 6 days rate | Mothers who received postnatal care within 6 days after delivery as proportion of deliveries in health facilities | NIDS |
| 11 | Couple year protection rate | Women protected against pregnancy by using modern contraceptive methods, including sterilisations, as proportion of female population 15-49 years. | NIDS |
| 12 | Termination of pregnancy 0-12 weeks rate | Pregnancies terminated in health facilities in the first 12 weeks of pregnancy as a proportion of total termination of pregnancies | NIDS |
| 13 | Antenatal client HIV re-test rate: retesting among positive HIV clients | Antenatal clients re-tested for HIV as proportion of antenatal clients tested negative for 1st HIV tests done during current pregnancy | NIDS |
| 14 | Average Ideal Clinic status (score) | We calculated the average Ideal Clinic score across facilities in a district or province, Ideal Clinic status is a score of 70% or more on assessment of the facility readiness to provide good quality of care along the following main dimensions 1. Administration 2. Integrated Clinical Services Management 3. Medicines, Supplies and Laboratory Services 4. Human Resources for Health 5. Support Services 6. Infrastructure 7. Health Information Management 8. Communication 9. District Health System Support 10. Implementing Partners and Stakeholders. | NIDS |
| 15 | Rural obstetric response under 40 minutes rate | Primary Obstetric calls responded to under 40 minutes in a rural area as a proportion of EMS (Emergency medical services) P1 rural obstetric calls total | NIDS |
| 16 | Urban obstetric response under 15 minutes rate | Primary Obstetric calls responded to under 15 minutes in an urban area as a proportion of EMS P1 urban obstetric calls total | NIDS |
| 17 | Domestic water compliance rate | Domestic bacteriological and chemical water samples taken from Water Services Authorities and water service intermediaries at a point of use that conform to the standards set out in SANS 241 for drinking water quality and safety as a proportion of water samples collected | NIDS |
| 18 | % women 15-49 who are literate | Proportion of women 15 - 49 who achieved grade 8 or more | GHS, CS, Census |
| 19 | % women 15-49 in households with adequate water infrastructure | Proportion of women 15-49 in households with adequate water supply infrastructure | GHS, CS, Census |
| 20 | % women 15 -49 with basic sanitation facility | Proportion of women 15-49 in households with basic sanitation facilities | GHS,CS, Census |
| 21 | % women 15-49 with access to electricity | Proportion of women 15-49 in households with access to electricity | GHS, CS,Census^a^ |
| 22 | % women 15-49 living in adequate housing | Proportion of women 15-49 living in households with “good” or “very good” wall, roof, and floor condition of the dwelling. | GHS |
| 23 | % women 15-49 living in formal housing | Proportion of women 15-49 in housing classified as formal housing (by Regional Development Plan (RDP) plan | GHS,CS, Census |
| 24 | % women 15 - 49 who have adequate food access | The mean proportion of women 15-49 in households that “never” had insufficient food, run out of money for food, cut the size of meals, skip a meal, or small variety of meals. | GHS,CS |
| 25 | Household Dietary Diversity Score | The Household Dietary Diversity Score by consumption of between 0-10 food groups, in households with women 15-49 years of age | GHS,CS |

GHS = General Household Survey, CS= Community Survey, Census^a^ = electricity access computed from source of energy for lighting
