# Supplementary material for: Development and testing of a composite index to monitor the continuum of maternal health service delivery at provincial and district level in South Africa
Source: PLoS One. 2021 May 25;16(5):e0252182. doi: 10.1371/journal.pone.0252182 (PMC8148336; doi:10.1371/journal.pone.0252182)
Supplement: Attachment — Submitted filename: MH_PONE-D-20-15418_reviewer.pdf [file pone.0252182.s002.pdf]

PLOS ONE

Development and testing of a composite index to monitor the continuum of maternal health service delivery at provincial and district level in South Africa

--Manuscript Draft--

**Manuscript Number:**	PONE-D-20-15418
**Article Type:**	Research Article
**Full Title:**	Development and testing of a composite index to monitor the continuum of maternal health service delivery at provincial and district level in South Africa
**Short Title:**	Composite index for continuum of maternal health service delivery
**Corresponding Author:**	Mamothena Carol Mothupi<br>University of the Western Cape<br>Bellville, SOUTH AFRICA
**Keywords:**	maternal health; continuum of care; social determinants of health; health service delivery; measurement theory; monitoring and evaluation
**Abstract:**	Introduction: The continuum of care is a recommended framework for comprehensive health service delivery for maternal health, and it integrates health system and social determinants of health. There is a current lack of knowledge on a measurement approach to be used to monitor performance on the framework. In this study we aim to develop and test a composite index for assessing the maternal health continuum in South Africa.<br>Materials and Methods: The composite index was computed as a geometric mean of four dimensions of adequacy of the continuum of care. Data was sourced from the district health information system, household surveys and the census. The index formula was tested for robustness when alternative inputs for indicators and standardization methods were used. The index was used to assess performance in service delivery in the North West province of South Africa, as well as its four districts over a five-year period (2013-2017). The index was validated by assessing associations with maternal health and other outcomes. And factor analysis was used to assess the statistical dimensions of the index.<br>Results: The provincial level index score increased from 62.3 in 2013 to 74 in 2017, showing general improvement in service delivery over time. The district level scores also improved over time, and our analysis identified areas for performance improvement. These include social determinants of health in some districts, and access and linkages to care in others. The provincial index was correlated with institutional maternal mortality rates (r_s =-0.90, 90% CI = (-1.00, -0.25)) and the Human Development Index (r=0.97, 95% CI = (0.63, 0.99). It was robust to alternative approaches including z-score standardization of indicators. Factor analysis showed three groupings of indicators for the health system and social determinants of health.<br>Conclusions: This study demonstrated the development and testing of a composite index to monitor and assess service delivery on the continuum of care for maternal health. The index was shown to be robust and valid, and identified potential areas for service improvement. A contextualized version can be tested in other settings within and outside of South Africa.
**Order of Authors:**	Mamothena Carol Mothupi
	Jeroen De Man
	Hanani Tabana
	Lucia Knight
**Additional Information:**	
**Question**	**Response**
**Financial Disclosure**<br><br>Enter a financial disclosure statement that describes the sources of funding for the	MM received funding to conduct this study. This work is based on the research supported by the South African Research Chairs Initiative of the Department of Science and Technology (https://www.dst.gov.za/) and National Research Foundation (https://www.nrf.ac.za/) of South Africa (Grant No. 82769). MM would also like to

work included in this submission. Review the submission guidelines for detailed requirements. View published research articles from *PLOS ONE* for specific examples.<br><br>This statement is required for submission and **will appear in the published article** if the submission is accepted. Please make sure it is accurate.<br><br>**Unfunded studies**<br>Enter: *The author(s) received no specific funding for this work.*<br><br>**Funded studies**<br>Enter a statement with the following details:<br>• Initials of the authors who received each award<br>• Grant numbers awarded to each author<br>• The full name of each funder<br>• URL of each funder website<br>• Did the sponsors or funders play any role in the study design, data collection and analysis, decision to publish, or preparation of the manuscript?<br>• **NO** - Include this sentence at the end of your statement: *The funders had no role in study design, data collection and analysis, decision to publish, or preparation of the manuscript.*<br>• **YES** - Specify the role(s) played.<br><br>* typeset	acknowledge funding from the South African Medical Research Council(https://www.samrc.ac.za/) and the Belgian Development Cooperation, through the Institute of Tropical Medicine(https://www.itg.be/) Antwerp. Any opinion, finding and conclusion or recommendation expressed in this material is that of the authors and not the funders. The funders did not have a role in the design of the study and collection, analysis, and interpretation of data and in writing the manuscript.
**Competing Interests**<br><br>Use the instructions below to enter a competing interest statement for this submission. On behalf of all authors, disclose any competing interests that could be perceived to bias this work—acknowledging all financial support and any other relevant financial or non-financial competing interests.<br><br>This statement **will appear in the published article** if the submission is accepted. Please make sure it is accurate. View published research articles from *PLOS ONE* for specific examples.	The authors have declared that no competing interests exist.

Powered by Editorial Manager® and ProduXion Manager® from Aries Systems Corporation

**NO authors have competing interests**<br><br>Enter: *The authors have declared that no competing interests exist.*<br><br>**Authors with competing interests**<br><br>Enter competing interest details beginning with this statement:<br><br>*I have read the journal's policy and the authors of this manuscript have the following competing interests: [insert competing interests here]*<br><br>* typeset	
**Ethics Statement**<br><br>Enter an ethics statement for this submission. This statement is required if the study involved:<br><br>• Human participants<br>• Human specimens or tissue<br>• Vertebrate animals or cephalopods<br>• Vertebrate embryos or tissues<br>• Field research<br><br>Write "N/A" if the submission does not require an ethics statement.<br><br>General guidance is provided below. Consult the submission guidelines for detailed instructions. **Make sure that all information entered here is included in the Methods section of the manuscript.**	N/A

Powered by Editorial Manager® and ProduXion Manager® from Aries Systems Corporation

**Format for specific study types**<br><br>**Human Subject Research (involving human participants and/or tissue)**<br>• Give the name of the institutional review board or ethics committee that approved the study<br>• Include the approval number and/or a statement indicating approval of this research<br>• Indicate the form of consent obtained (written/oral) or the reason that consent was not obtained (e.g. the data were analyzed anonymously)<br><br>**Animal Research (involving vertebrate animals, embryos or tissues)**<br>• Provide the name of the Institutional Animal Care and Use Committee (IACUC) or other relevant ethics board that reviewed the study protocol, and indicate whether they approved this research or granted a formal waiver of ethical approval<br>• Include an approval number if one was obtained<br>• If the study involved *non-human primates*, add *additional details* about animal welfare and steps taken to ameliorate suffering<br>• If anesthesia, euthanasia, or any kind of animal sacrifice is part of the study, include briefly which substances and/or methods were applied<br><br>**Field Research**<br><br>Include the following details if this study involves the collection of plant, animal, or other materials from a natural setting:<br>• Field permit number<br>• Name of the institution or relevant body that granted permission	
**Data Availability**<br><br>Authors are required to make all data underlying the findings described fully available, without restriction, and from the time of publication. PLOS allows rare exceptions to address legal and ethical concerns. See the PLOS Data Policy and FAQ for detailed information.	No - some restrictions will apply

Powered by Editorial Manager® and ProduXion Manager® from Aries Systems Corporation

A Data Availability Statement describing where the data can be found is required at submission. Your answers to this question constitute the Data Availability Statement and **will be published in the article**, if accepted.<br><br>**Important:** Stating 'data available on request from the author' is not sufficient. If your data are only available upon request, select 'No' for the first question and explain your exceptional situation in the text box.<br><br>Do the authors confirm that all data underlying the findings described in their manuscript are fully available without restriction?	
**Describe where the data may be found in full sentences. If you are copying our sample text, replace any instances of XXX with the appropriate details.**<br><br>• If the data are **held or will be held in a public repository**, include URLs, accession numbers or DOIs. If this information will only be available after acceptance, indicate this by ticking the box below. For example: *All XXX files are available from the XXX database (accession number(s) XXX, XXX.).*<br>• If the data are all contained **within the manuscript and/or Supporting Information files**, enter the following: *All relevant data are within the manuscript and its Supporting Information files.*<br>• If neither of these applies but you are able to provide **details of access elsewhere**, with or without limitations, please do so. For example:<br><br>*Data cannot be shared publicly because of [XXX]. Data are available from the XXX Institutional Data Access / Ethics Committee (contact via XXX) for researchers who meet the criteria for access to confidential data.*<br><br>*The data underlying the results presented in the study are available from (include the name of the third party*	District Health Information System data cannot be shared publicly because of Department of Health restrictions. Data are available from the North West Department of Health South Africa Institutional Data Access / Ethics Committee (contact via Dr. Frikkie Reichel FReichel@nwpg.gov.za) for researchers who meet the criteria for access to confidential data.<br><br>The data underlying the results presented in the study are available from (DataFirst – University of Cape Town and https://www.datafirst.uct.ac.za/dataportal/index.php/catalog).

and contact information or URL).<br>• This text is appropriate if the data are owned by a third party and authors do not have permission to share the data.<br><br>* typeset	
Additional data availability information:	

Powered by Editorial Manager® and ProduXion Manager® from Aries Systems Corporation

Manuscript Click here to download Manuscript Manuscript.docx

Development and testing of a composite index to monitor the continuum of maternal health service delivery at provincial and district level in South Africa

Authors: Mamothena Carol Mothupi[1], Jeroen De Man[2], Hanani Tabana[1], Lucia Knight[1]

Affiliations:

[1]School of Public Health, Faculty of Community and Health Sciences, University of the Western Cape, Cape Town, South Africa

[2]Centre for General Practice, Department of Primary and Interdisciplinary Care, University of Antwerp, Antwerp, Belgium

Corresponding Author:

mamothena@gmail.com (MCM)

Abstract

Introduction: The continuum of care is a recommended framework for comprehensive health service delivery for maternal health, and it integrates health system and social determinants of health. There is a current lack of knowledge on a measurement approach to monitor performance on the framework. In this study we aim to develop and test a composite index for assessing the maternal health continuum in South Africa

Materials and Methods: The composite index was computed as a geometric mean of four dimensions of adequacy of the continuum of care. Data was sourced from the district health information system, household surveys and the census. The index formula was tested for robustness when alternative inputs for indicators and standardization methods were used. The index was used to assess performance in service delivery in the North West province of South Africa, as well as its four districts over a five-year period (2013-2017). The index was validated by assessing associations with maternal health and other outcomes. And factor analysis was used to assess the statistical dimensions of the index.

Results: The provincial level index score increased from 62.3 in 2013 to 74 in 2017, showing general improvement in service delivery over time. The district level scores also improved over time, and our analysis identified areas for performance improvement. These include social determinants of health in some districts, and access and linkages to care in others. The provincial index was correlated with institutional maternal mortality rates (r_s=-0.90, 90% CI = (-1.00, -0.25)) and the Human Development Index (r=0.97, 95% CI = (0.63, 0.99). It was robust to alternative approaches including z-score standardization of indicators. Factor analysis showed three groupings of indicators for the health system and social determinants of health.

Conclusions: This study demonstrated the development and testing of a composite index to monitor and assess service delivery on the continuum of care for maternal health. The index was shown to be robust and valid, and identified potential areas for service improvement. A contextualised version can be tested in other settings within and outside of South Africa.

Introduction

Maternal health outcomes in South Africa (SA) remain poor despite national investments toward their improvement (1,2). The maternal mortality ratio (MMR) was estimated between 138 and 158 deaths per 100,000 live births in 2015(3). Pregnancy related and facility-based rates of maternal mortality are also high, estimated in 2016 at 536 per 100,000 and 135 per 100,000 respectively(2,4). The major causes of maternal mortality include HIV infection, obstetric haemorrhage, and hypertensive disorders(5). The prevailing challenges in maternal health in SA include: inequalities in health service access(6), poor coverage and quality of essential interventions(7), inadequate system wide improvements in quality of care(2) and weak community health services(2).

One of the key strategies to address maternal health challenges in SA has been to strengthen service provision at different levels of care, from the community to the district hospital (8–10). The continuum of care for maternal and child health is a public health framework for outlining the essential interventions and addressing service delivery challenges(11,12). The framework has been developed for low- and middle income countries (LMICs)(11) and adapted by national health system stakeholders to the South African context, as illustrated in Fig 1.

Fig 1. The continuum of care framework for maternal, new-born and child health in South Africa(8).

The framework outlines interventions from pre-pregnancy to childhood; the maternal health interventions encompass reproductive health, antenatal, delivery and postnatal care. The framework for SA also outlines "intersectoral factors", which represent social determinants of health such as housing, water and sanitation, and education(Fig 1). The implementation of the framework is expected to improve health outcomes by improving coverage and

comprehensiveness of services, mitigating duplication of resources, and improving integration of health services(11,13,14).

A crucial barrier to the implementation of the continuum of care framework in SA and many LMICs is the lack of a comprehensive monitoring tool for service delivery (15,16). The current discourse in SA focuses on the importance of integrated delivery of services, strengthening community health systems, and multisectoral collaboration to improve outcomes (1,9,13,17,18). Maternal death audits have emphasized the importance of referral linkages, women's empowerment and post-natal follow-ups to improve maternal health outcomes in the country (19). However, gaps remain in measuring community and social factors influencing maternal health outcomes(20). Assessment of the continuum of care requires consideration of a broad set of indicators beyond antenatal, birth and postnatal care(16).

Previous studies by these authors reviewed and evaluated available indicators for tracking services on the continuum of care for maternal health in South Africa (21,22). Another study by these authors proposed an analytical approach emphasizing assessment of access and utilization, quality of care, linkages of care and social determinants of health (16). Multidimensional assessment is often carried out with composite indices that summarize the performance of multiple interventions on the continuum. Composite indices have been used to track continuum of care performance at subnational and global levels(23–25), while a gap remains in broader integration of quality and social determinants of health (16,26). In this study we explore an approach to combine a broad set of indicators for maternal health interventions on the continuum of care through development of a composite index. We explore if the index can be used to assess service delivery at subnational levels in SA and the implications for future monitoring efforts to support implementation of the framework.

Methods

Setting

The North West province is one of the nine SA provinces and consists of four districts: Dr Kenneth Kaunda, Ngaka Modiri Molema, Dr Ruth Segomotsi Mompati, and Bojanala Platinum District Municipalities. This province is among the worst performers with regards to maternal health outcomes and health system indicators(27). However, the province was also one of the pioneers of primary health care quality improvement and health system strengthening initiatives, such as the Ward Based Outreach and the primary level Ideal Clinic realization programs (28)(29). Thus, the province was expected to have a broader set of available indicators across the continuum of care compared to others.

Design

We used a step-wise approach to develop and test the proposed index, based on current methodological guidelines (30,31). The main steps include: i) defining a theoretical/conceptual framework, ii) selection of variables/indicators, iii) imputation of missing data, iv) multivariate analysis, v) scaling of indicators, vi) weighting and aggregation, vii) checking for robustness, and viii) validation (30).

Conceptual framework

A critical interpretive synthesis of current measurement and monitoring approaches in LMICs found a gap in multi-dimensionality of sets of indicators currently used to assess the continuum of care (COC) for maternal health (16). The *adequacy construct* was therefore defined, which outlines four important dimensions to consider: 1) access and utilization of care; 2) quality of care; 3) linkages between levels and packages of care; and 4) social determinants of health (16) (Fig 2). The adequacy construct complements the COC framework by adding elements of quality of and linkages to care, and proposing that all four

dimensions be monitored, not just access and or utilization. Indicators of service delivery across all dimensions should therefore be sought from local data sources, with consideration for their relevance, feasibility and validity(21,22).

Fig 2 Dimensions and types of indicators used to develop the continuum of care index for maternal health

Selection of variables/indicators

A previous study assessed the suitability and measurement gaps of potential indicators for service delivery across the broad continuum in SA(21). These indicators were extracted for the North West province and districts for the period 2013-2017. Health service indicators were sourced from the National Indicator Data Set (NIDS) of the District Health Information System (DHIS). The DHIS is used to report and monitor facility level data for health services to support policy and planning(32). The DHIS provided indicators for access and utilization, linkages, and quality of care dimensions of the continuum of care.

Social determinant indicators were sourced from the annual *General Household Survey*(33) *(GHS) (2013-2017)*(33), *Census 2011*(34) and *Community Survey (CS) 2016* (35). The census and CS enabled assessment at district level, even though they offer fewer social determinants of health indicators than the GHS. The census and CS provided indicators of literacy, housing, access to electricity, water, and sanitation at the district level. Additionally, the CS also assesses dietary behaviour and empowerment, but this was not included in the final analysis of performance to allow district level comparison with the census indicator set. A description of all indicators used in this study is provided in S1 Table. Indicator data were extracted, cleaned, and analysed in MS Excel 2010, R v3.6.1 and STATA 14.0.

Imputation of missing data

Health service indicator data may be missing due to lack of services and under-performing systems for data collection and reporting. These are systematic issues that are considered to

affect availability of data for indicators completely at random. As such, we conducted single value imputation using the indicator value observed from the adjacent year(36). In the Results section we discuss the impact of the remaining data gaps on the index findings. Single value imputation was also applied to indicators from community survey and census to allow calculation of index at district level.

Multivariate analysis

We used exploratory factor analysis to assess dimensionality of the data, in order to compare the statistical and conceptual groupings of indicators (30). We assessed whether the data fitted the four dimensions of continuum of care proposed by the adequacy construct. The output of the exploratory factor analysis is assessed in the Results section.

Scaling of indicators

We conducted a linear transformation of indicator values on a scale between 0 and 100 (37) (Equation 1).

$$\text{Indicator score} = \text{Ideal Score-}\left|\left(\text{Target-Performance}\right)*100\right| \quad (1)$$

The **indicator score** is calculated on a scale between 0 – 100; the **ideal score** is the maximum attainable score, which is a 100; the **target** is the ideal performance of the indicator; and **performance** is the observed value of the indicator during a given time period. Targets may consist of a range of values and in such a case we calculated the median score to represent indicator performance. Targets were also based on national policy documents and global technical or scientific guidelines (30,31,37). Targets were set to the conservative maximum (100%) where guidelines were unavailable. The difference between target and observed performance is multiplied by 100 because indicators are originally measured as percentages/proportions. Using targets for performance improves the meaningfulness of the index and its’ role in policy discourse(38).

Weighting and aggregation

The comprehensive continuum of care for maternal health index (C_3MH index) was computed as a geometric mean of equally weighted sub-indices reflecting the four adequacy dimensions. We chose equal weighting since this was estimated the most reasonable approach, and evidence on the relative importance of each sub-index is lacking (30,31,39).

$$C_3\text{MH index} = (\text{Access to care}\cdot\text{Linkages}\cdot\text{Quality of Care}\cdot\text{Social Determinants of Health})^{1/4} \quad (2)$$

Simple indices, based on arithmetic and geometric means, can be robust and give valuable information about public health or health system performance (24,25,39–41). Unlike the arithmetic mean, the geometric mean allows for a degree of non-compensation of performance of one indicator by another(30,31). Each sub-index (e.g. access to care) was also formulated as a geometric mean of its indicator scores.

$$\text{Sub index score} = (\text{Indicator}_a\cdot\text{Indicator}_b\cdot\text{Indicator}_c\cdots\text{Indicator}_n)^{1/n} \qquad (3)$$

Where *a, b, c* are individual indicators and *n* = number of indicators within the sub-index.

Validity and Robustness

We ran sensitivity analyses comparing index performance with different indicator combinations and normalization methods (42). We tested if z-score standardization leads to a shift in district ranks (30). Index performance was also compared after removal of indicators that were considered outliers (performed close to 100 %), missing data, or indicators that could be represented by a proxy (e.g. syphilis treatment measured with one indicator instead of the three across the treatment cascade, see S1 Table Indicators 4-6). Index aggregation by arithmetic and geometric mean was also compared. We assessed the median absolute difference in district ranks, and its inter-quartile range, testing alternative approaches to index formulation (42). External validation of the index was conducted by exploring its relationship with indicators of public health performance and maternal health outcomes, particularly the

Human Development Index (HDI) and maternal mortality rates (37,43,44). Confidence intervals for correlations were calculated by bootstrapping methods in R v3.6.1.

Results

Performance at provincial level

In the North West province, we combined 12 indicators of access and utilization to care, two quality of care, two linkages of care and 9 social determinants of health indicators to measure the C3MH index (Table 1).

Table 1: The continuum of care for maternal health index, sub- indices and indicators for North West Province, South Africa, in the period 2013-2017.

		Indicator Performance					Indicator Scores				
**Indicators**	**Targets**	**2013**	**2014**	**2015**	**2016**	**2017**	**2013**	**2014**	**2015**	**2016**	**2017**
Cervical cancer screening coverage	100%	58%	62%	66%	66%	69%	**58**	**62**	**66**	**66**	**69**
Antenatal 1st visit before 20 weeks rate	100%	48%	53%	59%	64%	64%	**48**	**53**	**59**	**64**	**64**
Antenatal 1st visit coverage	100%	78%	79%	75%	76%	78%	**78**	**79**	**75**	**76**	**78**
Syphilis positive pregnant female receive Benz-penicillin 1st dose rate	100%	na	na	57%	57%	78%	**na**	**na**	**57**	**57**	**78**
Syphilis positive pregnant female receive Benz-penicillin 2nd dose rate	100%	na	na	60%	60%	57%	**na**	**na**	**60**	**60**	**57**
Syphilis positive pregnant female receive Benz-penicillin 3rd dose rate	100%	na	na	57%	57%	50%	**na**	**na**	**57**	**57**	**50**
Antenatal client starts on antiretroviral therapy rate	100%	63%	88%	89%	93%	93%	**63**	**88**	**89**	**93**	**93**
Delivery by Caesarean section rate	5-15%	18%	21%	22%	21%	24%	**92**	**89**	**88**	**89**	**86**
Delivery in facility rate	100%	65%	67%	69%	69%	72%	**65**	**67**	**69**	**69**	**72**
Mother postnatal visit within 6 days rate	80-100%	75%	76%	71%	73%	77%	**85**	**86**	**81**	**83**	**87**
Couple year protection rate	50-100%	42%	54%	50%	59%	55%	**67**	**75**	**75**	**75**	**75**
Termination of pregnancy 0-12 weeks rate	100%	97%	96%	95%	95%	96%	**97**	**96**	**95**	**95**	**96**
Access sub-index							***70.9***	***76.0***	***71.5***	***72.5***	***74.1***
Antenatal client HIV re-test rate	100%	47%	64%	78%	100%	100%	***47***	***64***	***78***	***100***	***100***
Average ideal clinic status (score)	70-100%	na	55%	55%	65%	66%	***na***	***70***	***70***	***80***	***81***
Quality sub-index							***47***	***66.9***	***73.9***	***89.4***	***90.0***
Emergency rural obstetric response under 40 minutes rate	75-100%	na	na	na	61%	61%	***na***	***na***	***na***	***74***	***74***

Emergency urban obstetric response under 15 minutes rate	75-100%	na	na	na	41%	41%	*na*	*na*	*na*	*54*	*54*
Linkages sub-index							*na*	*na*	*na*	*62.7*	*62.7*
Domestic water compliance rate	100%	72%	53%	62%	76%	63%	*72*	*53*	*62*	*76*	*63*
% women 15-49 who are literate	100%	83%	84%	83%	83%	82%	*83*	*84*	*83*	*83*	*82*
% women 15-49 in households with adequate water infrastructure	100%	82%	82%	78%	80%	80%	*82*	*82*	*78*	*80*	*80*
% women 15 -49 with basic sanitation facility	100%	71%	69%	70%	71%	71%	*71*	*69*	*70*	*71*	*71*
% women 15-49 living in adequate housing	100%	52%	44%	48%	54%	55%	*52*	*44*	*48*	*54*	*55*
% women 15-49 living in formal housing	100%	85%	85%	82%	82%	83%	*85*	*84*	*82*	*82*	*83*
% women 15-49 with access to electricity	100%	95%	95%	94%	94%	95%	*95*	*95*	*94*	*94*	*95*
% women 15 - 49 who have adequate food access	100%	62%	62%	62%	64%	64%	*62*	*62*	*62*	*64*	*64*
Mean Household Dietary Diversity Score (women 15-49) (converted to 100)	100%	62	61	62	62	62	*62*	*61*	*62*	*62*	*62*
SDoH index score							*72.6*	*68.6*	*69.9*	*73.0*	*71.8*
CoC (maternal health) Index							*62.3*	*70.4*	*71.7*	*73.8*	*74.0*

SDoH= social determinants of health; CoC= continuum of care.

The C_3MH index at the provincial level ranged from 62.3 in 2013 to 74 in 2017, showing a general trend of improvement (see table 1). The two sub-indices that substantially contributed to this increase were:

- *Improvement in access and utilization of care indicators*, particularly cervical cancer screening, timely antenatal care, and ante-retr[illegible]al drug provision.
- *Improvement in facility performance on quality of care measures*. The quality sub-index improved from 66.9 in 2014 to 90 in 2017. However, this sub-index is only based on two indicator[illegible]ne of which represents a drastic improvement in HIV program processes. A gap exists in maternal health care safety and patient experience of care indicators for measurement of quality in the North West province.

Little overall improvement was made in the social determinants of health during that period, which may point to a slow pace of development in the province. Data was unavailable for the

period (2013-14) to monitor treatment of sexually transmitted illness (syphilis), emergency obstetric transport (2013-2015), and quality of care (Ideal Clinic) (2013-2014).

Monitoring performance at district level

There was an overall improvement in the index at district level over the period 2013-2017, as illustrated in Fig 3.

Fig 3 Comprehensive continuum of care index (C3MHindex) scores by districts over a five-year period 2013- 2017

Overall, Dr Ruth Segomotsi Mompati (RSM) district performed better than other districts on the index, while Bojanala Platinum performed generally poorer. We also compared sub-index performance to demonstrate effect on overall scores, using 2016 as a reference year (Fig 4).

Fig 4 Sub-index and C_3MH index scores by districts in 2016. BN = Bojanala Platinum District, KK = Dr Kenneth Kaunda District Municipality, RSM = Dr Ruth Segomotsi Mompati District Municipality, NMM = Ngaka Modiri Molema District Municipality, SDOH = Social determinants of health.

In 2016, Dr Kenneth Kaunda district scored relatively higher than other districts on the social determinants of health and quality of care sub-indices. But due to poor performance on access and linkages of care, the district scored second best in overall performance in 2016. Comparatively, the Ruth Segomotsi Mompati district had relatively high scores across sub-indices, and thus ranked highest in 2016. Thus, the balance of good performance across all sub-indices improved the overall index.

Robustness

There was no significant difference in district ranks between index scores calculated with linearly scaled indicators (our method) and z-score standardization (r_s=0.83, 95% CI =(0.49-0.95)) (Table 2). There was also no significant difference between index scores when geometric and arithmetic aggregation techniques were used (r_s =0.95, 95% CI=(0.78-0.99)). The median absolute difference in index rankings at district level when linear and z-score

standardization were compared was 2 ranks with an interquartile range (IQR) of 0-3. There was no difference in rankings (IQR= 0-1) observed at district level when indices computed with arithmetic and geometric means were compared.

Table 2 Spearman rank correlation between alternatives for indicator standardization and aggregation at district level

	**Base case**	**z-score (districts)**	**arithmetic mean**
**Base case**	1.00		
**z-score**	0.83	1.00	
**arithmetic mean (d)**	0.95	0.84	1.00

Base case is based on linear scaling (our method) and geometric mean aggregation.

All the index values across alternative indicator selections were highly correlated (Table 3).

Table 3 Spearman rank correlation coefficients of index values when dropping one indicator at a time to compute index

	**Base case (all)**	**No syphilis 2&3**	**No termination**	**No syphilis & no termination**
**Base case (all)**	1.00			
**No syphilis 2&3**	0.98	1.00		
**No termination**	0.99	0.97	1.00	
**No syphilis & no termination**	0.98	0.99	0.98	1.00

Validation

The C_3MH index had a positive correlation **(**r=0.972, 95% CI = (0.63, 0.99)) with the Human Development Index (HDI) in the North West province for the period 2013-2017. The HDI measures healthy life outcomes, education and standard of living(45). The index also increased with decreasing rates of institutional maternal mortality at the provincial level (r_s= -0.90, 90% CI = (-1.00, -0.25)). The correlation between the index and iMMR at district level, was not statistically significant (r= -0.13, 95% CI = (-0.58, 0.39). There are no data for HDI scores at district level to allow comparisons with the COC index.

Results of multivariate analysis

Parallel analysis in exploratory factor analysis suggested one main underlying factor for the data (Fig 5), although a three-factor model may be possible.

Fig 5 Parallel analysis scree plot for indicators of the continuum of care for maternal health in North West province South Africa

A one factor model accounted for 0.52 proportion of variance of the data. A three-factor model accounted for cumulative variance of >0.9 : the majority of factor 1 indicators related to the health system or facility based care, factor 2 contained both health system and social determinants of health, and factor 3 contained social determinants (Table 4). A two-factor model accounted for 0.72 cumulative proportion of variance of the data but did not reveal any informative conceptual groupings – all factor loadings were relatively high for one factor. The variables for linkages of care and one variable for quality of care were also not included in the results of the model due to missing data.

Table 4 Exploratory factor analysis of the items of the continuum of care service delivery framework

	Factor 1	Factor 2	Factor 3
Cervical screening	**0.93**	-0.25	0.20
Timely antenatal visit	**0.90**	-0.33	0.31
ARTs during antenatal care	**0.95**	-0.25	-0.21
Caesarean section delivery	**0.91**	-0.08	-0.02
Delivery in facility	**0.91**	-0.12	0.25
Couple year protection rate	**0.86**	-0.09	-0.05
HIV retest rate	**0.91**	-0.24	0.33
Adequate food	**0.77**	0.14	0.47
Ante natal care coverage	-0.06	**0.99**	-0.14
Postnatal visit	0.18	**0.98**	0.06
Timely pregnancy termination	**-0.59**	**0.75**	0.15
Water infrastructure	-0.45	**0.83**	-0.13
Type of housing	-0.52	**0.78**	-0.35
Electricity access	-0.33	**0.91**	-0.23
Water safety compliance	-0.23	-0.26	**0.79**
Literacy	-0.37	0.02	**-0.77**
Sanitation	0.20	0.08	**0.98**
Housing condition	0.25	0.01	**0.97**
Household dietary diversity	-0.04	-0.45	**0.86**

Notes: Extraction method – ordinary least squared/minres; Rotation – varimax; Loading larger than 0.5 are in bold.

Discussion

This study demonstrated the development and testing of a comprehensive and multidimensional index to assess the continuum of care for maternal health at subnational levels in SA. The C_3MH index measured health and non-health sector components of service delivery for maternal health, as guided by the continuum of care framework for SA(8). The multi-sectoral perspective of the index is increasingly important in current public health and health system performance assessment (15,46,47). The index was comprehensive in that it allowed monitoring of myriad interventions, summarized through four sub- indices representing dimensions of the continuum of care. The comprehensive and multisectoral character of the index contrasts with the "silo" or vertical program approach that singles out single interventions to address maternal health outcomes(48).

Our findings suggest that the index can be used as a monitoring tool to compare subnational performance over time, and as a basis for recommendations on areas of service delivery improvement. For instance, our findings show that improvements in access and linkages to care will enhance Dr Kenneth Kaunda district's overall score and improve its ranking. On the other hand, in Dr Ruth Segomotsi Mompati district, poor performance on the social determinants of health affected the index. Thus, while the C_3MH index can be used to compare and rank districts, an analysis of sub- indices indicates areas that may proportionally affect overall performance. The relevance and utility of sub-components of composite indices for public health policy and action is important to consider as well, beyond the monitoring application of the overall index(31,38).

Our findings also indicate that the index was robust and not much influenced by outlying scores for specific indicators. In other words, the index values the parts differently than the whole, as well as good performance over several indicators and not just a few outlying values. Further research is needed to compare the index to other comprehensive standards for

the integrated care approach it seems to reflect. Alternative methods for computing the index and standardising the indicators produced comparable results. The simple geometric approach allows future integration of missing data, while maintaining the conceptual grounding of the index. Our approach also accommodates the expected refinements of indicators over time (49); the index should undergo recurrent assessment and validation to remain useful (50). Additionally, exploratory factor analysis indicates a distinction between at least 2 factors, with one factor covering health systems indicators and the other factor(s) social determinants of health. This reflects the multidimensional nature of the index and underlines the need to include social determinants as a dimension of continuum of care in maternal health.

Limitations

This was a case study of five subnational geographical areas over a five-year period. We recommend more research across other provinces/districts to allow further comparison. In other countries, the same approach using a comprehensive set of available indicators can be used to develop a contextualised version of the index. The composition of the index in this study was affected by gaps in data availability common in the SA health system (51). We recommend health information system improvements in monitoring and availability of data so that better estimates of the index can be made in the future. In addition, the lack of comparability of provincial and district level associations between the index and maternal outcomes warrants further investigation. There may be systemic issues with maternal mortality estimation in the country(52), and we also recommend use of the GHS as a source of data at district level. Other indicators could also be considered as proxies based on their shown reciprocity with maternal health indicators, such as neonatal and child health outcome[illegible]4,25,53,54).

Conclusion

This study shows the feasibility to monitor and assess service delivery for the continuum of care for maternal health using indicators from different sectors with a composite index. The index allows monitoring of performance over time and across geographical areas. From our analyses, we concluded the index to be robust and valid, with potential to guide policy and planning to improve maternal health outcomes and service delivery from a multisectoral perspectiv[illegible] More comprehensive monitoring of social determinants at district level and health information system strengthening can further improve and extend the use of this index. The index is amenable for testing with data from different South African and international contexts.

Acknowledgements

References

1. Rispel L. Analysing the progress and fault lines of health sector transformation in South Africa. South African Heal Rev. 2016;17–24.

2. Pillay Y, Barron P. On the path to reach the SDG targets: Decreasing maternal and child mortality in South Africa. South Africa Med J. 2018;3(Suppl 1):S2–3.

3. Day C, Gray A, Ndlovu N. Health and related indicators. South African Heal Rev [Internet]. 2018;139–250. Available from: http://hst.org.za/uploads/files/sahr2006.pdf

4. Department of Health, Statistics South Africa, South African Medical Research Council. South Africa Demographic and Health Survey 2016 [Internet]. Pretoria, South Africa; 2019. Available from: https://dhsprogram.com/pubs/pdf/FR337/FR337.pdf

5. Department of Health. Saving Mothers 2014-2016 : Seventh triennial report on confidential enquiries into maternal deaths in South Africa : Executive Summary.

Pretoria, South Africa; 2016.

6. Wabiri N, Chersich M, Shisana O, Blaauw D, Rees H, Dwane N. Growing inequities in maternal health in South Africa : a comparison of serial national household surveys. BMC Pregnancy Childbirth [Internet]. 2016;16(256). Available from: http://dx.doi.org/10.1186/s12884-016-1048-z

7. Bhardwaj S, Pattinson R, Sa F, Kauchali S, Sa F, Dlamini N, et al. Implementation of strategies to improve programme effectiveness lead to an improvement in maternal and child health outcomes in South Africa. South Africa Med J. 2018;108(March):44–52.

8. Bradshaw D, Chopra M, Kerber K, Lawn J, Moodley J, Pattinson R, et al. Every Death Counts: Saving the Lives of Mothers, Babies and Children in South Africa [Internet]. Every Death Counts: Saving the Lives of Mothers, Babies and Children in South Africa. Cape Town, South Africa; 2008 [cited 2018 Jun 9]. Available from: http://www.mrc.ac.za/sites/default/files/attachments/2016-06-30/edcrptfinal.pdf

9. Department of Health South Africa. Strategic Plan for Maternal , Newborn , Child and Women ' s Health (MNCWH) and Nutrition in South Africa: 2012 -2016. 2012;

10. Statistics South Africa. Millennium Development Goals 5: Improve Maternal Health, 2015. 2015. 34 p.

11. Kerber KJ, de Graft-Johnson JE, Bhutta ZA, Okong P, Starrs A, Lawn JE. Continuum of care for maternal, newborn, and child health: from slogan to service delivery. Lancet. 2007;370(9595):1358–69.

12. WHO. The World Health Report 2005: Make Every Mother and Child Count. Geneva, Switzerland; 2005.

13. Bamford L. Maternal, Newborn and Child Health. In: Padarath A, English R, editors. South African Health Review 2012/2013. 2012/2013. Durban, South Africa: Health Systems Trust; 2012. p. 49–66.

14. Chopra M, Daviaud E, Pattinson R, Fonn S, Lawn JE. Saving the lives of South Africa's mothers, babies, and children: can the health system deliver? Lancet. 2009 Sep 5;374(9692):835–46.

15. Department of Health. Strategic Plan for Maternal, Newborn, Child and Women's Health (MNCWH) and Nutrition in South Africa: 2012 -2016. Pretoria, South Africa; 2012.

16. Mothupi M, Knight L, Tabana H. Measurement approaches in continuum of care for maternal health: a critical interpretive synthesis of evidence from LMICs and its implications for the South African context. BMC Health Serv Res. 2018;18(539):1–9.

17. Chola L, Pillay Y, Barron P, Tugendhaft A, Kerber K, Hofman K. Cost and impact of scaling up interventions to save lives of mothers and children: taking South Africa closer to MDGs 4 and 5. Glob Health Action [Internet]. 2015;8:27265. Available from: http://www.pubmedcentral.nih.gov/articlerender.fcgi?artid=4408314&tool=pmcentrez&rendertype=abstract

18. Mayosi BM, Lawn JE, van Niekerk A, Bradshaw D, Abdool Karim SS, Coovadia HM. Health in South Africa: changes and challenges since 2009. Lancet. 2012 Dec 8;380(9858):2029–43.

19. National Committee for Confidential Enquiry into Maternal Deaths. Saving Mothers 2014-2016 : Seventh triennial report on confidential enquiries into maternal deaths in South Africa: Executive Summary. Pretoria, South Africa; 2016.

20. Moodley J, Pattinson RC, Fawcus S, Schoon MG, Moran N, Shweni PM. The confidential enquiry into maternal deaths in South Africa: a case study. BJOG. 2014 Sep;121 Suppl:53–60.

21. Mothupi MC, Knight L, Tabana H. Review of health and non-health sector indicators for monitoring service provision along the continuum of care for maternal health. BMC Res Notes [Internet]. 2020; Available from: https://www.researchsquare.com/article/rs-12586/v3

22. Mothupi MC, Knight L, Tabana H. Improving the validity, relevance and feasibility of the continuum of care framework for maternal health in South Africa: a thematic analysis of experts' perspectives. Heal Res Policy Syst . 2019;

23. Kumar C, Singh PK, Rai RK. Coverage gap in maternal and child health services in India: Assessing trends and regional deprivation during 1992-2006. J Public Heal (United Kingdom). 2013;35(4):598–606.

24. Boerma JT, Bryce J, Kinfu Y, Axelson H, Victora CG. Mind the gap:equity and trends in coverage of maternal, newborn, and child health services in 54 countdown countries. Lancet [Internet]. 2008;371:1259–67. Available from: ?http://www.thelancet.com/journals/lancet/article/PIIS0140-6736(08)60560-7/fulltext?

25. Wehrmeister FC, Restrepo-mendez M, Franca VA, Victora G. Summary indices for monitoring universal coverage in maternal and child health care. Bull World Health Organ. 2016;94(November):903–12.

26. Graham WJ, Varghese B. Quality, quality, quality: Gaps in the continuum of care. Lancet [Internet]. 2012;379(9811):e5–6. Available from: http://dx.doi.org/10.1016/S0140-6736(10)62267-2

27. Department of Health. Annual Performance Plan 2018/19 - 2020/21. Pretoria, South Africa; 2018.

28. Schneider H, Besada D, Sanders D, Daviaud E, Rohde S. Ward-based primary health care outreach teams in South Africa: developments, challenges and future directions. In: Rispel L, Padarath A, editors. South African Health Review 2018. 2018th ed. Durban, South Africa: Health Systems Trust; 2018. p. 59–66.

29. Hunter J, Asmall S, Ravhengani NM, Chandran TM, Tucker J-M, Mokgalagadi Y. The Ideal Clinic in South Africa: progress and challenges in implementation. In: Padarath A, Barron P, editors. South African Health Review 2017. 2017th ed. Durban, South Africa: Health Systems Trust; 2017. p. 12.

30. OECD. Handbook on Constructing Composite Indicators: Methodology and User Guide. Paris, France: OECD Publications; 2008.

31. Profit J, Typpo K V, Hysong SJ, Woodard LD, Kallen MA, Petersen LA. Improving benchmarking by using an explicit framework for the development of composite indicators : an example using pediatric quality of care. Implement Sci. 2010;1–10.

32. Department of Health. District Health Management Information System (DHMIS) Policy. Pretoria, South Africa; 2011.

33. Statistics South Africa. Open Data Portal: General Household Survey [Internet]. Data First - University of Cape Town; Available from: https://www.datafirst.uct.ac.za/dataportal/index.php/catalog/central

34. Statistics South Africa. Open Data Portal: Census 2011 South Africa [Internet]. Data First - University of Cape Town; Available from: https://www.datafirst.uct.ac.za/dataportal/index.php/catalog/485

35. Statistics South Africa. Nesstar [Internet]. 2017 [cited 2018 Dec 16]. Available from: http://interactive.statssa.gov.za:8282/webview/

36. Mikkelsen EM, Cronin-fenton D, Kristensen NR, Pedersen L. Missing data and multiple imputation in clinical epidemiological research. Clin Epidemiol. 2017;9:157–66.

37. Booysen F. An overview and evaluation of composite indices of development. Soc Indic Res. 2002;59(2):115–51.

38. Barclay M, Dixon-woods M, Lyratzopoulos G. The problem with composite indicators. BMJ Qual Saf. 2019;28:338–44.

39. Noble M, Barnes H, Wright G, McLennan D, Avenell D, Whitworth A, et al. The South African Index of Multiple Deprivation 2001 at the Datazone Level. Pretoria; 2009.

40. Boerma T, Requejo J, Victora CG, Amouzou A, George A, Agyepong I, et al. Countdown to 2030: tracking progress towards universal coverage for reproductive, maternal, newborn, and child health. Lancet. 2018;391(10129):1538–48.

41. Stern S, Wares A, Orzell S, O'Sullivan P. SOCIAL PROGRESS INDEX 2014: Methodological Report. 2014.

42. Hogan DR, Stevens GA, Hosseinpoor AR, Boerma T. Monitoring universal health coverage within the Sustainable Development Goals : development and baseline data for an index of essential health services. Lancet Glob Heal [Internet]. 2018;6(2):e152–68. Available from: http://dx.doi.org/10.1016/S2214-109X(17)30472-2

43. Bolarinwa O. Principles and methods of validity and reliability testing of

questionnaires used in social and health science researches. Niger Postgrad Med J [Internet]. 2015;22(4):195. Available from: http://www.npmj.org/text.asp?2015/22/4/195/173959

44. Bhattacherjee A. Social Science Research: Principles, Methods and Practices. 2nd ed. Vol. 3, Scholar Commons Textbooks Collection. USF Tampa Library Open Access Collections at Scholar Commons; 2012.

45. United Nations Development Program. Technical Notes: Calculating the Human Development Indices - Graphical Presentation. Human Development Report 2016. New York: United Nations Development Program; 2016.

46. Begg K, Mamdoo P, Dudley L, Andrews G, Engelbrecht J, Lebese L. Development of a national strategic framework for a high-quality health system in South Africa. In: Rispel LC, Padarath A, editors. South African Health Review 2018. 2018th ed. Durban, South Africa: Health Systems Trust; 2018. p. 77–86.

47. Maina I, Wanjala P, Soti D, Kipruto H, Droti B, Boerma T. Using health-facility data to assess subnational coverage of maternal and child health indicators, Kenya. Bull World Health Organ. 2017 Oct 1;95(10):683–94.

48. Horwood C, Haskins L, Vermaak K, Phakathi S, Subbaye R, Doherty T. Prevention of mother to child transmission of HIV (PMTCT) programme in KwaZulu-Natal , South Africa : an evaluation of PMTCT implementation and integration into routine maternal , child and women ’ s health services. Trop Med Int Heal. 2010;15(9):992–9.

49. Department of Health. National Indicator Data Set Data Elements. Pretoria, South Africa; 2019.

50. Benova L, Moller A, Moran AC. “ What gets measured better gets done better ”: The

landscape of validation of global maternal and newborn health indicators through key informant interviews. PLoS One. 2019;14(11):1–16.

51. English R, Masilela T, Barron P, Schonfeldt A. Health Information Systems in South Africa. South African Heal Rev. 2011;81–9.

52. Damian DJ, Njau B, Lisasi E, Msuya SE, Boulle A. Trends in maternal and neonatal mortality in South Africa: A systematic review. Syst Rev. 2019;8(1):1–13.

53. Wilunda C, Putoto G, Riva DD, Manenti F. Assessing Coverage , Equity and Quality Gaps in Maternal and Neonatal Care in Sub- Saharan Africa : An Integrated Approach. PLoS One. 2015;10(5):1–16.

54. Dettrick Z, Gouda HN, Hodge A, Jimenez-Soto E. Measuring quality of maternal and newborn care in developing countries using demographic and health surveys. PLoS One. 2016;11(6):1–21.

Supporting Information

S1 Table. Indicators used for measurement of the continuum of care index in North West province and districts 2013-2017

Figures and Tables

Fig 1. The continuum of care framework for maternal, new-born and child health in South Africa.

Fig 2 Dimensions and types of indicators used to develop the continuum of care index for maternal health

Fig 3 Comprehensive continuum of care index (C3MHindex) scores by districts over a five-year period 2013- 2017

Fig 4 Sub-index and C3MH index scores by districts in 2016. BN = Bojanala Platinum District, KK = Dr Kenneth Kaunda District Municipality, RSM = Dr Ruth Segomotsi Mompati District Municipality, NMM = Ngaka Modiri Molema District Municipality, SDOH = Social determinants of health.

Fig 5 Parallel analysis scree plot for indicators of the continuum of care for maternal health in North West province South Africa

Table 1: The continuum of care for maternal health index, sub- indices and indicators for North West Province, South Africa, in the period 2013-2017.

Table 2 Spearman rank correlation between alternatives for indicator standardization and aggregation at district level

Table 3 Spearman rank correlation coefficients of index values when dropping one indicator at a time to compute index

Table 4 Exploratory factor analysis of the items of the continuum of care service delivery framework

Figure 1 Click here to download Figure Fig1.tif

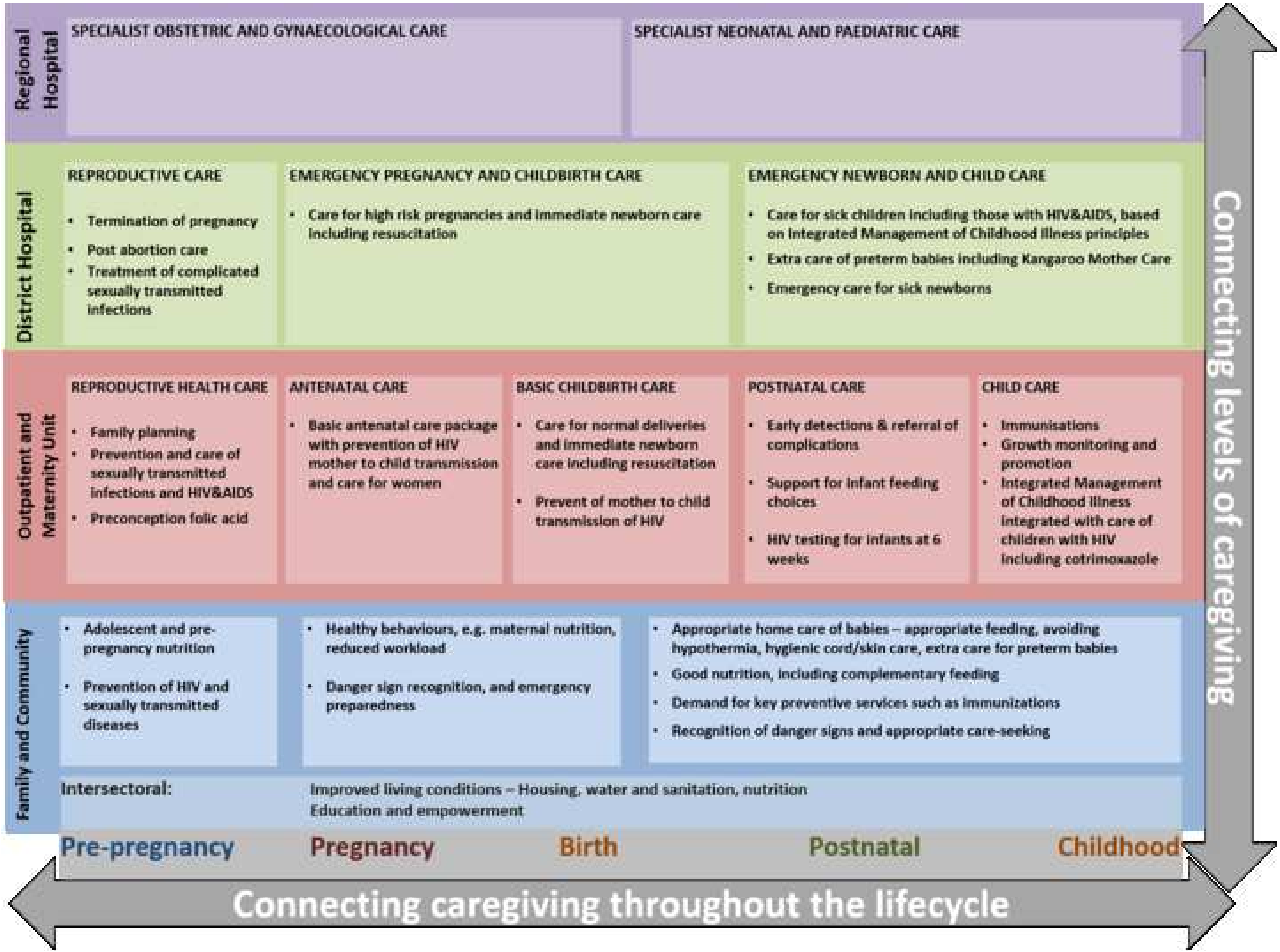

Figure 3 Click here to download Figure Fig3.tif

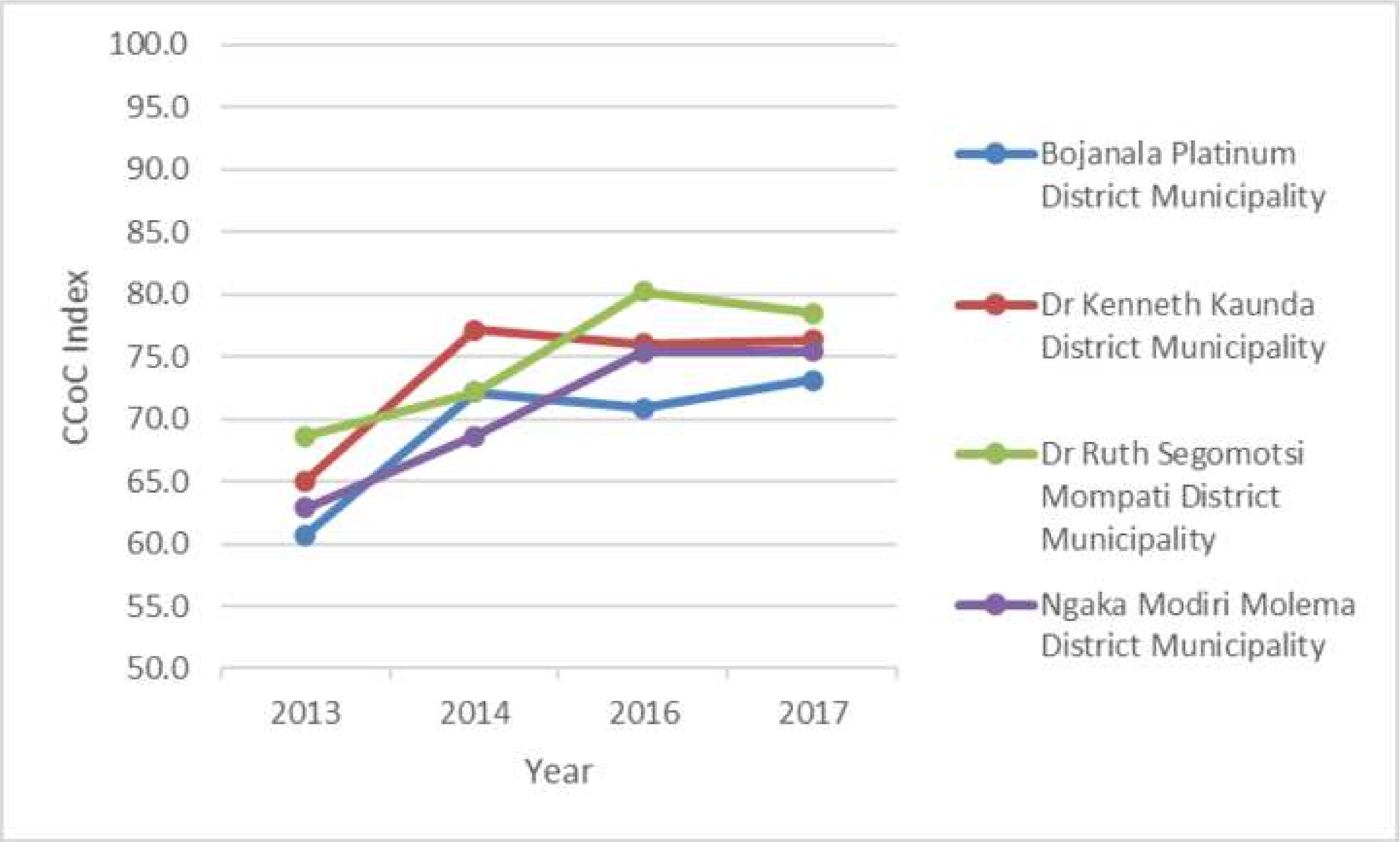

Figure 4

Click here to download Figure Fig4.tif

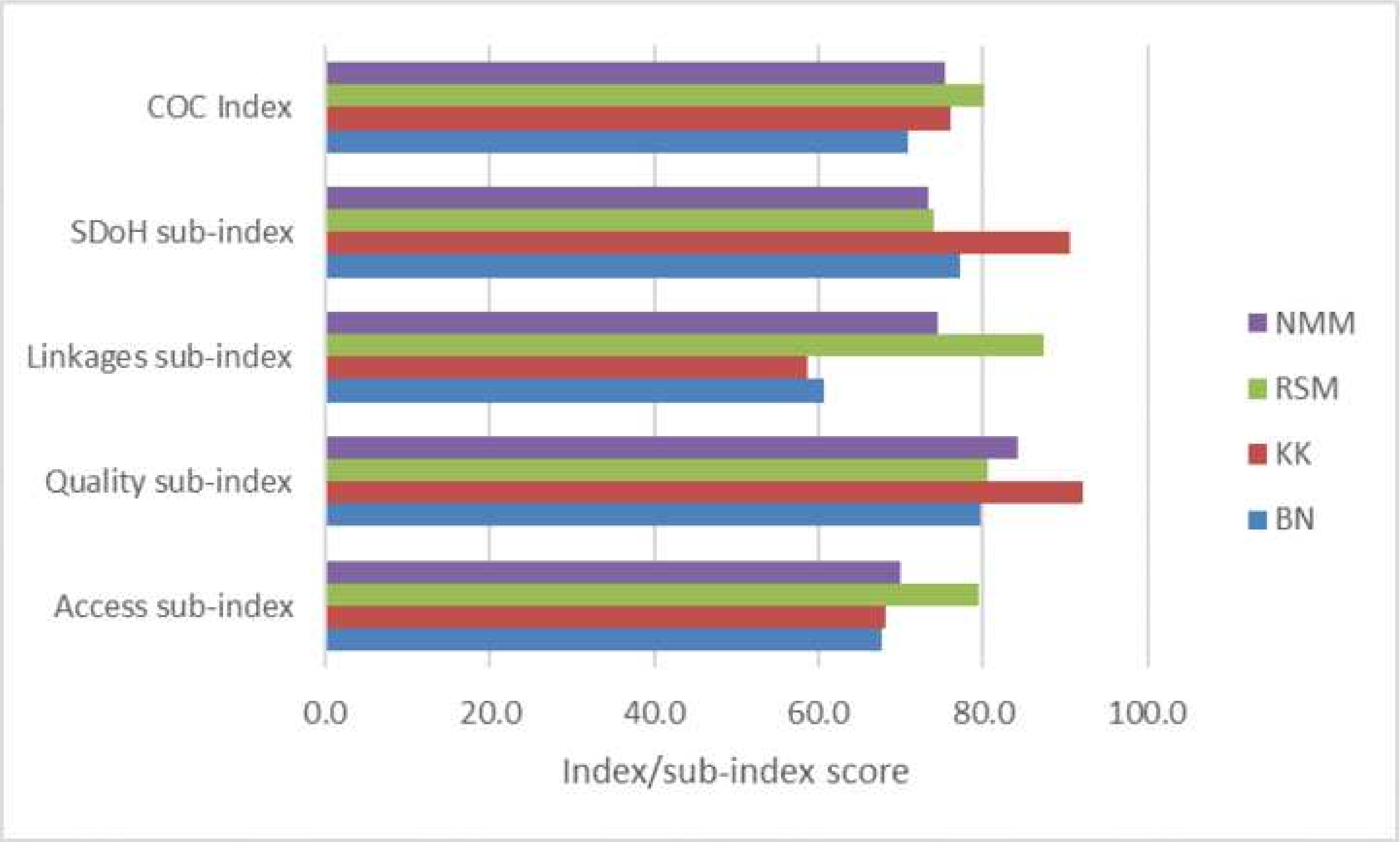

Figure 5

Click here to download Figure Fig5.tif

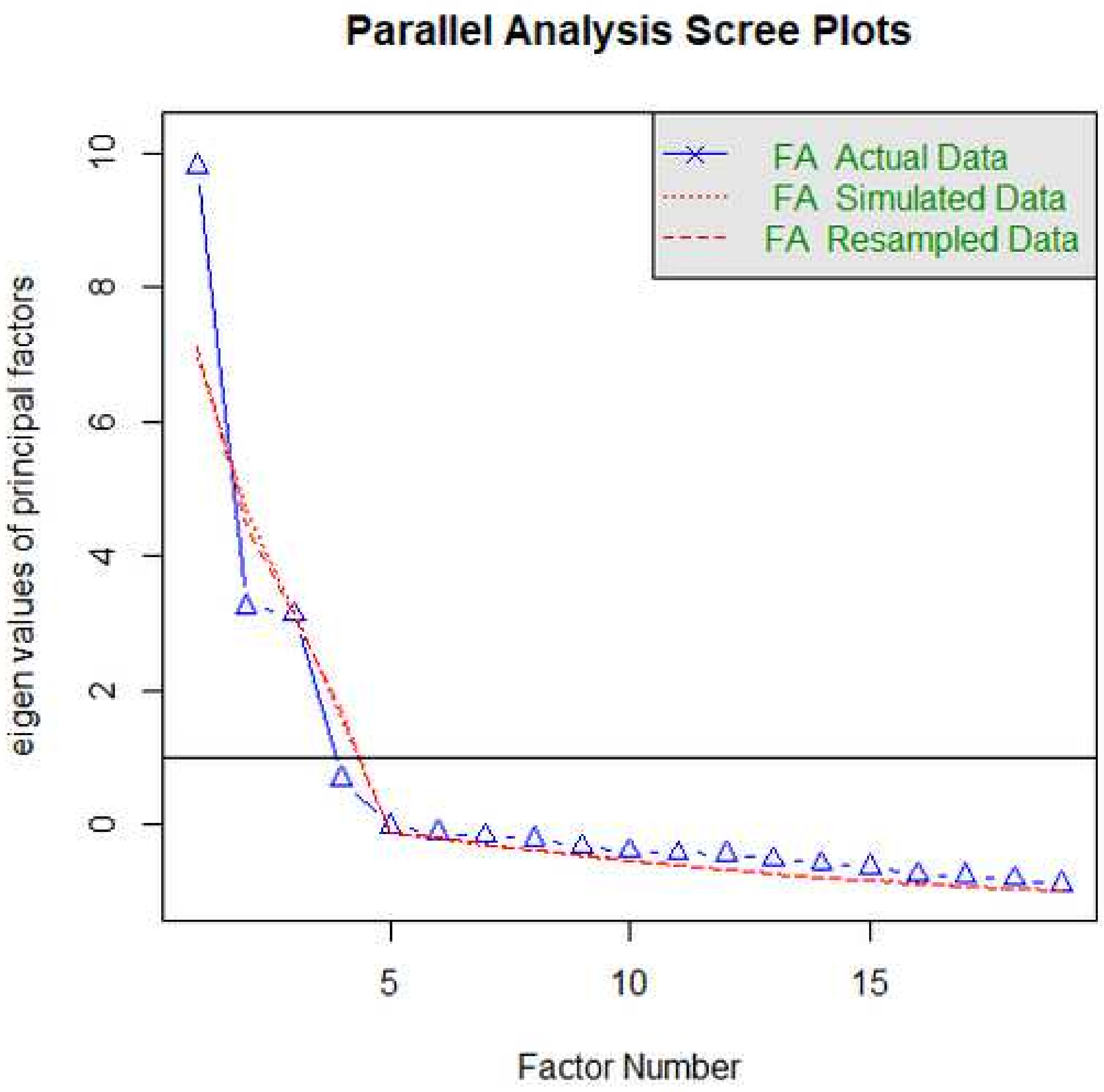

Figure 2

Click here to download Figure Fig2.tif

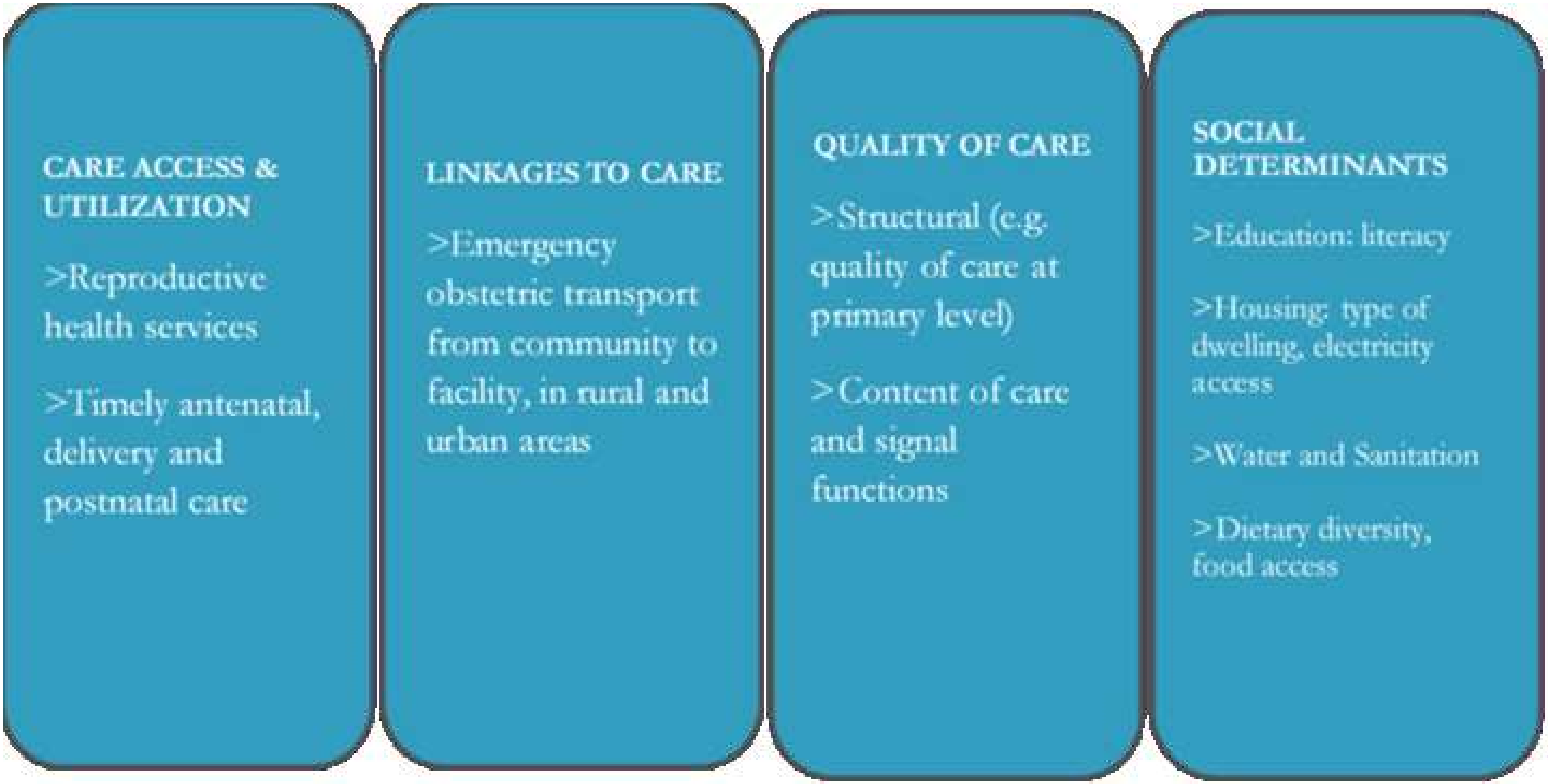

Click here to access/download
Supporting Information
S1 Table.docx

Click here to access/download
Data Review URL
http://www.datafirst.uct.ac.za/dataportal/index.php/catalo
g/485

Click here to access/download
Data Review URL
http://www.datafirst.uct.ac.za/dataportal/index.php/catalog/611

Click here to access/download

Data Review URL

http://www.datafirst.uct.ac.za/dataportal/index.php/catalog/StatsSA#_r=1589794622042&collection=&country=&dtype=&from=1947&page=1&ps=&sk=general+household+survey&sort_by=nation&sort_order=&to=2019&topic=&view=s&vk=
