# Supplementary material for: Development and testing of a composite index to monitor the continuum of maternal health service delivery at provincial and district level in South Africa
Source: PLoS One. 2021 May 25;16(5):e0252182. doi: 10.1371/journal.pone.0252182 (PMC8148336; doi:10.1371/journal.pone.0252182)
Supplement: Attachment — Submitted filename: Development and testing of a composite index to monitor the continuum of maternal.docx [file pone.0252182.s003.docx]

**Development and testing of a composite index to monitor the continuum of maternal**

**health service delivery at provincial and district level in South Africa**

Reviewer’s comments

This is a very important study and potentially adds to the literature and can be very useful to programme managers. It’s a brave attempt especially given issues related to data availability and quality in South Africa and in many LMICs.

In their review of the literature I would request the authors to pay more attention to issues related to quality of care and outcomes (these issues are reflected in my notes in the text of their manuscript).

There are areas of concern with the methodology that I wish to flag for the considerations of the authors. The author should be add a critical review of the use of linear transformation (see Greco et al, 2019 for example). In addition, the selection of indicators used (especially those from the DHIS) and their decision not to include mortality (maternal and neonatal mortality) as outcomes should be clearly stated. The latter is only noted in the section on limitations of the study. Finally, the quality of the DHIS data (in additional to imputation of missing data) should be reflected on.

On the practical use of the index – the authors should inform the reader if the study and its approach, results and conclusions were discussed with the local managers and if any of their recommendations were adopted/implemented. I am aware that this is not usually requested by reviewers but practical application of such studies are important to improve quality of care.

I would therefore encourage the authors to strengthen the aspects reflected above and as reflected in my notes in their manuscript.

Yogan Pillay

3 March 2021
